# Integration of GWAS and Co-Expression Network Analysis Identified Main Genes Responsible for Nitrogen Uptake Traits in Seedling Waxy Corn

**DOI:** 10.3390/genes16020126

**Published:** 2025-01-23

**Authors:** Chunmei Luo, Huixue Dai, Shuaiqiang Liang, Han Zhao, Ling Zhou

**Affiliations:** 1 College of Agriculture, Anhui Science and Technology University, Chuzhou 233100, China; 18605861790@163.com; 2 Nanjing Vegetables Research Institute, Nanjing 210042, China; 3 Jiangsu Academy of Agricultural Sciences, Institute of Genetic Resources and Biotechnology, Nanjing 210014, Chinazhaohan@jaas.ac.cn (H.Z.)

**Keywords:** waxy corn, nitrogen uptake, GWAS, co-expression network

## Abstract

**Background/Objectives:** Waxy corn has a unique taste and flavor that a majority of consumers love, and the market application prospect is broad. Nitrogen plays an important role in the growth and development of waxy corn. Exploring the key genes that affect nitrogen absorption can lay a foundation for improving the quality of waxy corn. **Methods:** In this study, a total of 534 local waxy corn inbred lines were used to perform genome-wide association studies (GWAS) to mine the significant Quantitative Trait Nucleotides (QTNs) for nitrogen content of waxy corn at seedling stage in two different environments. The Weighted Gene Co-Expression Network Analysis (WGCNA) nitrogen response co-expression network was also constructed to explore the differences of gene expression patterns and the co-expression relationship between transcription factors and functional genes to find candidate genes significantly associated with nitrogen uptake in waxy corn. **Results:** A total of 97 significant associations (LOD-value ≥ 3) were detected between SNPs and nitrate content traits under single and multi-environment conditions. Fifty-four candidate genes were identified around the significant SNPs in about a 20 Kb region. Combined with nitrogen response differential co-expression network analysis, 17 out of the 54 candidate genes were identified in the nitrogen response module, among which 4 main genes (*Zm00001d029012*, *Zm00001d034035*, *Zm00001d007890*, and *Zm00001d045097*) were repeatedly detected in multiple environments. **Conclusions:** This study jointly identified four stable and heritable candidate genes involved in the nitrogen metabolism process through GWAS and co-expression network analysis. The results of this study provide theoretical guidance for further elucidating the genetic mechanism of nitrogen efficiency in waxy corn and breeding new germplasm of waxy corn.

## 1. Introduction

The amylopectin content of waxy corn can reach up to 99%, giving it a distinctive soft and sweet texture. Waxy corn has higher nutritional value compared to sweet corn and is more easily absorbed by the human body, making it a favorite among consumers. As a result, waxy corn has significant market potential on a global scale [1]. China has abundant varieties of waxy corn. Previous studies have found that Southwest China, especially Yunnan and its surrounding areas, is the center of origin of waxy corn in China [2]. Although waxy corn originated in China and has obvious resource advantages, the research on waxy corn breeding was not reported until the 1970s. Compared with Western countries, the research on waxy corn in China started late, and there are fewer researchers specializing in waxy corn breeding [3].

China has the largest planting area of waxy corn in the world [1]. Nitrogen is significantly associated with the quality and yield of waxy corn. The contents of protein and soluble sugar in grain of waxy corn increased first and then decreased with the increase in nitrogen application [4]. In the middle and late growth and development of waxy corn, scientific application of nitrogen fertilizer can improve the grain quality of waxy corn [5]. It is of great significance to reduce nitrogen consumption and breed novel corn varieties by exploring candidate genes for nitrogen efficient absorption and developing germplasm resources and new varieties of nitrogen efficient glutinous corn. In order to analyze the genetic mechanism of nitrogen absorption and metabolism-related traits in maize, many studies have been conducted. For example, *ZmDof1* is the first transcription factor found to be related to the regulation of nitrogen uptake in maize [6]. The expression of *ZmDof1* in rice can enhance the absorption and assimilation of carbon and nitrogen in the low-nitrogen environment. Ge Ming et al. analyzed the gene regulatory network constructed from the pan-transcriptome data of 503 maize inbred lines and identified *ZmNLP5* as a key node. *ZmNLP5* plays a crucial role in the nitrogen uptake and assimilation process of maize under nitrogen starvation [7]. Moreover, some transcription factors, such as MADS-box transcription factors, were reported to be closely related to nitrogen uptake and assimilation in rice and *Arabidopsis*. In maize, the first MADS-box transcription factor, *Zmm28*, was found to be closely related to nitrogen utilization, and overexpression of *Zmm28* significantly increased maize yield [8]. Liu et al. identified a locally nitrogen-responsive MADS-box transcription factor, *ZmTMM1*, which is primarily expressed in roots. *ZmTMM1* regulates root configuration under varying nitrogen levels and influences the absorption of nitrate by roots [9]. Du et al. analyzed the nitrogen response-conserved modules of maize and sorghum through gene co-expression networks and identified the evolutionally conserved nitrogen regulatory transcription factor *ZmNIGT1* in maize and sorghum; *ZmNIGT1* is a negative regulatory factor and plays an important role in optimizing nitrogen nutrition metabolism [10]. The exploration of the regulatory factors related to nitrogen absorption and metabolism is primarily conducted through omics and reverse-genetics studies. Further investigation is required to uncover additional regulatory factors in maize using forward genetics methods.

Nitrogen-use efficiency (NUE) is mainly determined by the interaction of absorption, transport, and assimilation. As the first step of nitrogen assimilation in plants, nitrogen uptake is a typical complex quantitative trait controlled by multiple genes. In recent years, GWAS has been widely used to find genes associated with NUE traits, as a powerful tool to link genotype–phenotype information [11]. Julia et al. (2017) evaluated the root length (TRL) and low nitrogen tolerance index (LNTI) of 64 tropical maize inbred lines under high- and low-nitrogen conditions and identified 7 SNPs that were significantly correlated with NUE traits using GWAS [12]. Ma et al. (2020) conducted GWAS, using 226 maize double haploid populations as materials and 61,634 SNPs for genotyping, and studied the genetic basis of root system architecture (RSA) under two nitrogen levels. Under high-nitrogen and low-nitrogen conditions, 33 and 51 significant SNP and trait associations were detected. Some of the linkage disequilibrium (LD) regions of SNPs overlapped with the RSA and N-response quantitative trait loci (QTL) regions detected in previous studies, covering 8 candidate genes [13]. Ndlovu et al. (2022) conducted an optimal- and low-nitrogen field trial in Kenya and South Africa, assessing the association mapping populations of 410 maize inbred lines and 4 parental populations, and genotyping with 259,798 SNP markers. Under low-nitrogen stress, 42 SNPs related to grain-quality traits were identified by GWAS. These significant SNPs were associated with 51 candidate genes [14]. These identified sites related to NUE traits may have significant effects on the root length, root structure, and grain quality of maize, but the sites related to NUE in glutinous maize have not been reported.

Co-expression network analysis is an important method for studying the relationship between gene expression patterns and functions using massive biological data. In the co-expression network, genes with similar expression patterns are clustered into the same module [15]. It is generally believed that genes with the same expression pattern may have the same functions, thus facilitating speculation about the function of unknown genes. At present, co-expression network analysis has been used to reveal the molecular mechanism of rice seedling root to enhance nitrogen-use efficiency by regulating nitrogen uptake and utilization, carbon metabolism, root growth and development, and plant hormone-related genes [16]. There have also been studies conducted to extract information from gene co-expression networks of leguminous crops in order to enhance the understanding of plant nitrogen-fixation pathways [17]. There have also been lots of reports about drought resistance, disease resistance, cold tolerance, and heavy metal tolerance in maize through co-expression networks [18,19,20,21]. In waxy corn, the team led by Yan Jianbing has revealed the complex genetic and molecular mechanisms underlying the formation of waxy corn flavor through genomic, metabolomic, and co-expression network analyses [22]. However, there are few studies on nitrogen uptake analysis of waxy corn using co-expression networks.

In this study, a total of 534 diverse populations of waxy corn germplasm resources were used as experimental materials. The accumulation of nitrate in their leaves was evaluated through phenotype analysis, GWAS, and the construction of co-expression networks. The objective of this study was to investigate nitrate accumulation in maize and identify potential sites and candidate genes that may influence nitrate content. The findings from this study could provide a theoretical and scientific foundation for understanding nitrogen uptake and metabolism, as well as related regulatory networks in maize, with potential applications in breeding.

## 2. Materials and Methods

### 2.1. Plant Materials

The experimental materials included Jingnuo 210, Sichuan purple waxy corn, Guanglingxiang waxy corn, Caihua waxy corn, local red waxy corn, golden waxy corn, East purple waxy corn, white waxy corn, etc. These waxy corn germplasm resources with wide distribution, rich genetic background, and significant morphological differences from all over China were provided by the Jiangsu Provincial Crop Germplasm Resources medium-term bank “http://jagis.jaas.ac.cn (accessed on 11 October 2020)”, with a total of 534 (Supplementary Table S1).

### 2.2. Phenotype Determination and Analysis

In this experiment, the nitrate content at the seedling stage was studied under two different planting environments. In total, 534 waxy corn germplasm resources were planted in the Hainan (E1) base and Liuhe base (E2) of Jiangsu Academy of Agricultural Sciences in 2021 and 2022, respectively. In two separate experimental plots, seeds were sown in 7 rows, with 80 rows in each plot. Each row was sown with 10 plump seeds, with a row spacing of 60 cm and a row length of 5 m. Conventional field management measures, including weed control, topdressing, irrigation, and drainage, were carried out properly. Compound fertilizer (200 kg·hm^−2^ nitrogen, 800 kg·hm^−2^ phosphate, and 150 kg·hm^−2^ potassium) was applied before sowing. Samples were collected when the corn plants reached the four-leaf stage. The nitrate content in maize plants was determined using Wang Xuekui’s method [23], which employs the salicylic acid–sulfuric acid method. A UV–visible light spectrophotometer was used to measure the absorption value at 410 nm, and the nitrate content was calculated based on the nitrate standard curve.

### 2.3. Genotype Analysis

The improved CTAB method [24] was used to extract genomic DNA from 534 waxy corn samples, and genotyping was conducted by BGI Sequencing Company. It is suitable for GWAS, genetic map construction, and heterosis population division of maize. The original genotype data were filtered using Plink1.9 software [25], and SNPs with low quality and a minimum allele frequency of less than 0.05 were excluded. Afterward, the missing genotype data were imputed using TASSEL5.2.28 [26] software, resulting in high-quality SNPs for further analysis.

### 2.4. Genome-Wide Association Analysis and Candidate Gene Prediction

The ADMIXTURE 1.3 software [27], developed by Alexander et al., was used to determine the optimal number of subgroups [28]. Ten independent repeated operations were carried out for each K value. When the cross-validation error (CV Error) was at its lowest, the corresponding K value represented the optimal number of subgroups. We used TBtools V2.056 software [29] for visual mapping.

For genome-wide association analysis, the IIIVmrMLM1.0 software [30] was utilized in conjunction with population structure (Q matrix), and kin relationship (K matrix) was used to perform GWAS of genotype data and nitrate content using a mixed linear model (MLM). A LOD-value of ≥3 was used as the criterion for determining significant SNPs [31], and visualization of the Manhattan map and Quantile–Quantile Plot was performed by the IIIVmrMLM package in R-4.3.2 software [32].

The GWAS test was associated with a significant nitrate content of SNP loci, using SnpEff tools [33] according to the maize B73 reference genome “https://www.maizegdb.org/ (accessed on 1 December 2022)” for significant SNP loci gene annotations.

### 2.5. Nitrogen-Response Expression Analysis of the Candidate Genes

The experimental material used was the B73 inbred line. Nitrogen treatment was performed after two weeks of nitrogen starvation. The statistical data related to the leaf expression changes in maize treated with 10 mM KNO_3_ at 0 min, 10 min, 20 min, 30 min, 60 min, and 90 min.

To construct the nitrogen response co-expression network, the transcriptome was first analyzed, with each sample of corn leaves derived from three independent biological replicates. The total RNA was extracted using TRIzol reagent according to the manufacturer’s instructions. After the quality control process, the library was constructed and sequenced as instructed by Berry Genomics company (Beijing, China). Analysis of RNA-seq data was based on previous studies [34]. The value of FPKM (fragments per kilobase of exon per million fragments mapped) was calculated using Cufflinks (v2.1.1) software [35]. Two-way ANOVA is performed using a custom function in the R programming language to identify transcripts that exhibit differential expression across various stages. Then, 5% of the models were corrected using the false discovery rate (FDR) [36]. According to the WGCNA program [37], co-expression networks were constructed, and module detection was carried out based on the selected model and developmental-stage transcripts (*p*-value ≤ 0.05).

The SEA (Singular Enrichment Analysis) tool of online software AgriGO V2.0 was used to further classify the candidate genes with log2 fold change (FC) > 1 and (FC) < −1 in corn according to statistical significance (*p*-value ≤ 0.05).

## 3. Results

### 3.1. Identification of Germplasm Resources and Construction of Variation Map of Local Waxy Corn

To evaluate the phenotypic variations among the experimental materials, we investigated nitrate content in multiple environments, including Hainan (2021, E1) and Liuhe (2022, E2), for the association panel. As can be seen from Table 1, the nitrate content of local waxy corn materials planted in the E1 and E2 areas shows no significant change at the seedling stage, and it essentially follows a normal distribution within the population.

**Figure 1:**
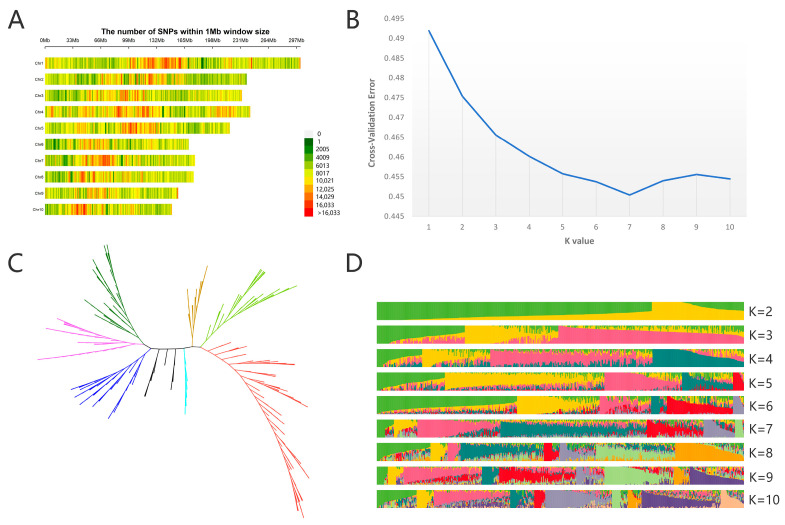
Genetic relationship analysis of 534 waxy corn germplasm resources. (**A**) High-density and high-precision variation map of waxy corn, (**B**) cross-check error curve, (**C**) phylogenetic tree of 534 maize inbred lines, and (**D**) population number.

Using Next-Generation Sequencing (NGS) technology, 109,178,752 mutation sites with high PIC (Polymorphism Information Content) were detected from 534 samples (Table 2). The R package (CMplot) was used to create a comprehensive map of DNA variations across the entire genome of waxy corn (Figure 1A). SNPs’ screening and filtering were carried out using strict criteria to ensure the accuracy of SNP sites. In addition, variation sites were very widely distributed across the genome, and the frequency of variation detected in the centromere region of chromosomes was low. The use of variation maps can provide theoretical and data support for exploring highly polymorphic variation sites. This can further assist in designing and developing highly specialized and highly polymorphic molecular markers.

### 3.2. Genome-Wide Association Analysis of Nitrogen Uptake Traits

By filtering out SNP markers obtained from simplified genome sequencing of natural populations, 1,768,120 SNP sites were identified for subsequent analysis. The optimal subpopulation number is calculated using the ADMIXTURE software. When the cross-validation error (CV error) is at its lowest, the optimal subpopulation number is the K value at that time (Figure 1B,D). In total, 534 maize inbred lines are divided into seven subpopulations by calculation: Subgroup I (17 members), Subgroup II (60 members), Subgroup III (181 members), Subgroup IV (6 members), Subgroup V (104 members), Subgroup VI (76 members), and Subgroup VII (90 members). In addition, the neighbor-joining method in MEGA7 software was used to build the phylogenetic tree [38] (Figure 1C). The results of the evolutionary tree analysis indicated that the genetic diversity within the seven subpopulations is relatively high, making them suitable for association analysis.

**Figure 2:**
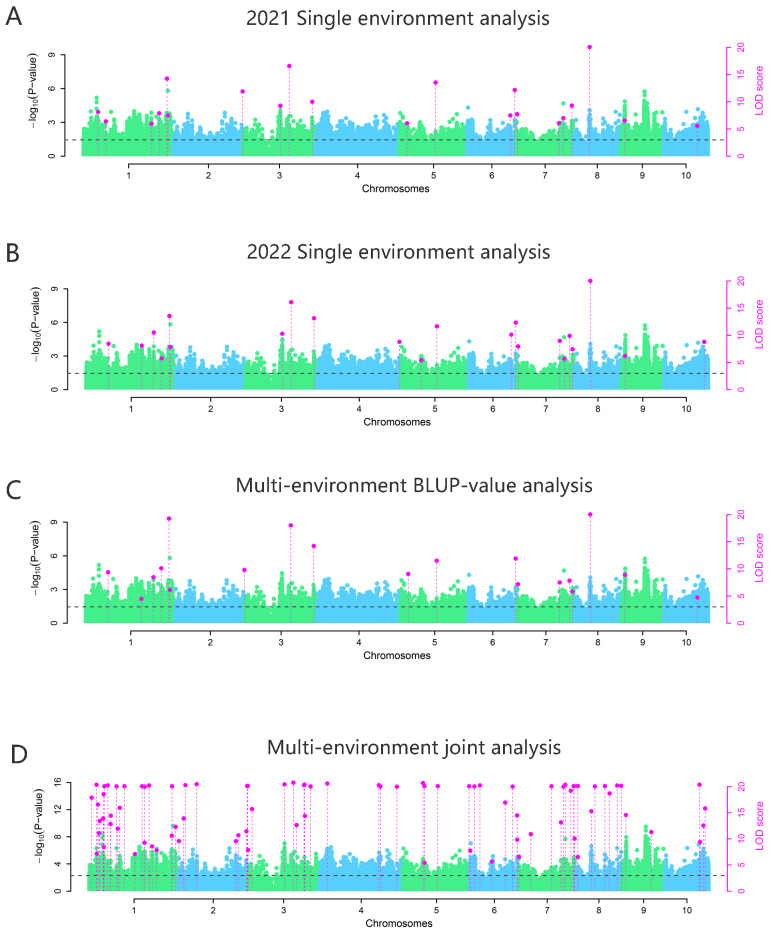
GWAS analysis of nitrogen absorption traits of 534 waxy corn cultivars. (**A**) The 2021 planting in Sanya, Hainan. (**B**) The 2022 planting in Jiangsu, Liuhe. (**C**) Multi-environment BLUP value analysis. (**D**) Multi-environment joint analysis. Dots above the dotted line represent SNPS significantly associated with nitrogen uptake traits, while blue and green blocks are used to distinguish between different chromosomes.

Based on 1,768,120 high-quality SNP markers, the IIIVmrMLM method [39] was used to conduct a GWAS on nitrate content traits of 534 waxy corn germplasm resources from two different environments at the seedling stage. An LOD-value ≥ 3 was used as the criterion for a significant association. The results showed that, in the single environment (E1) of Sanya Base in Hainan Province in December 2021, there were 21 SNP loci significantly associated with nitrate content in maize. In the single environment (E2) of Liuhe base in Jiangsu Province in June 2022, there were 22 SNP loci significantly associated with nitrate content in maize. A total of 20 SNPs significantly associated with nitrate content in maize were excavated by epistatic association analysis with BLUP values across multiple environments. A total of 86 SNPs significantly associated with nitrate content in maize were identified through joint-association analysis of multiple environmental data, with the SNPs on chromosome 1 being the most prevalent (Figure 2). The ANNOVAR software (http://annovar.openbioinformatics.org/en/latest/user-guide/download/) was used to annotate the genes of 97 significant QTNs (LOD-value ≥ 3). We found that 54 candidate genes were closely linked to nitrogen uptake traits at significant QTNs (within the range of 20 Kb). The results revealed that 54 candidate genes were detected near the QTN (approximately 20 Kb region) and were closely associated with markers. Among these, 17 candidate genes were consistently identified in multiple environments (Table 3).

### 3.3. Transcriptome Co-Expression Network Construction

**Figure 3:**
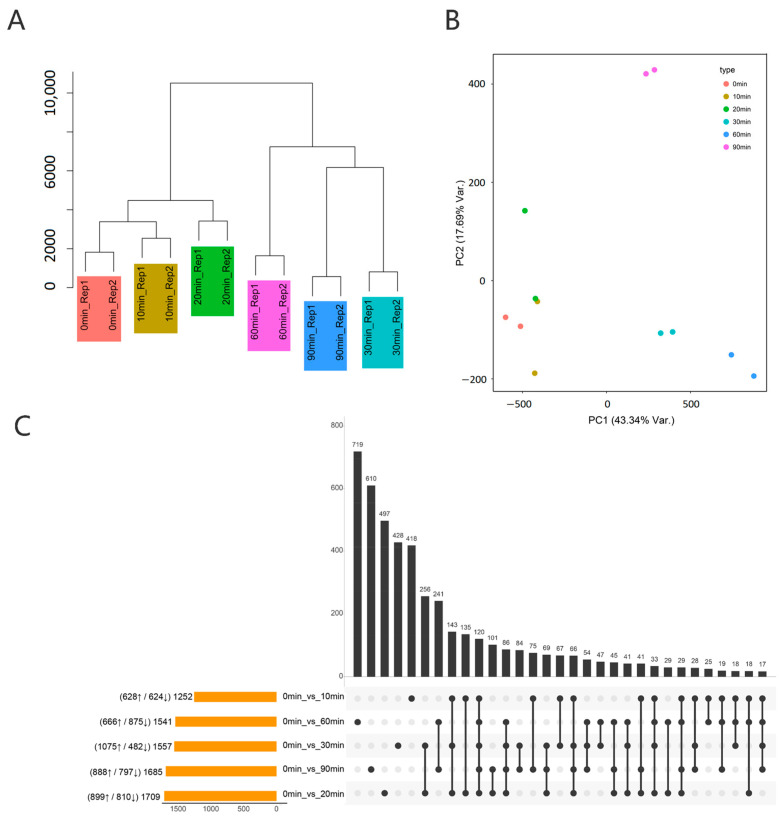
Transcriptome sample clustering and differentially expressed genes under the treatment of six time gradients of nitrate salt environment. (**A**) Clustering of transcriptome samples at different time points. (**B**) Principal component clustering analysis. (**C**) Number of differentially expressed genes at different time points.

In this study, B73 material was treated with a nitrate environment (sufficient nitrogen, SN) at six time gradients. Transcriptome sequencing was performed using two biological replicates at different time points (0, 10, 20, 30, 60, and 90 min). The transcriptome transcripts per million (TPM) value was used for clustering and Principal Component Analysis (PCA) dimensionality reduction analysis, and the deviation from the mean was eliminated. The results showed that different repeat samples at the same time point could be better clustered together, as shown in Figure 3A,B. Based on the read count value of each transcript, the differentially expressed genes among different samples were analyzed using the EdgeR package. With a *p*-value < 0.05 and |log2FC| > 1 as thresholds, a total of 4559 significantly differentially expressed genes (DEGs) were identified under nitrogen treatment. The number of differentially expressed genes in response to nitrogen varied at different time points. The number of genes up-regulated by nitrogen fluctuated between 628 and 1075, while the number of genes down-regulated fluctuated between 482 and 875 (Figure 3C).

**Figure 4:**
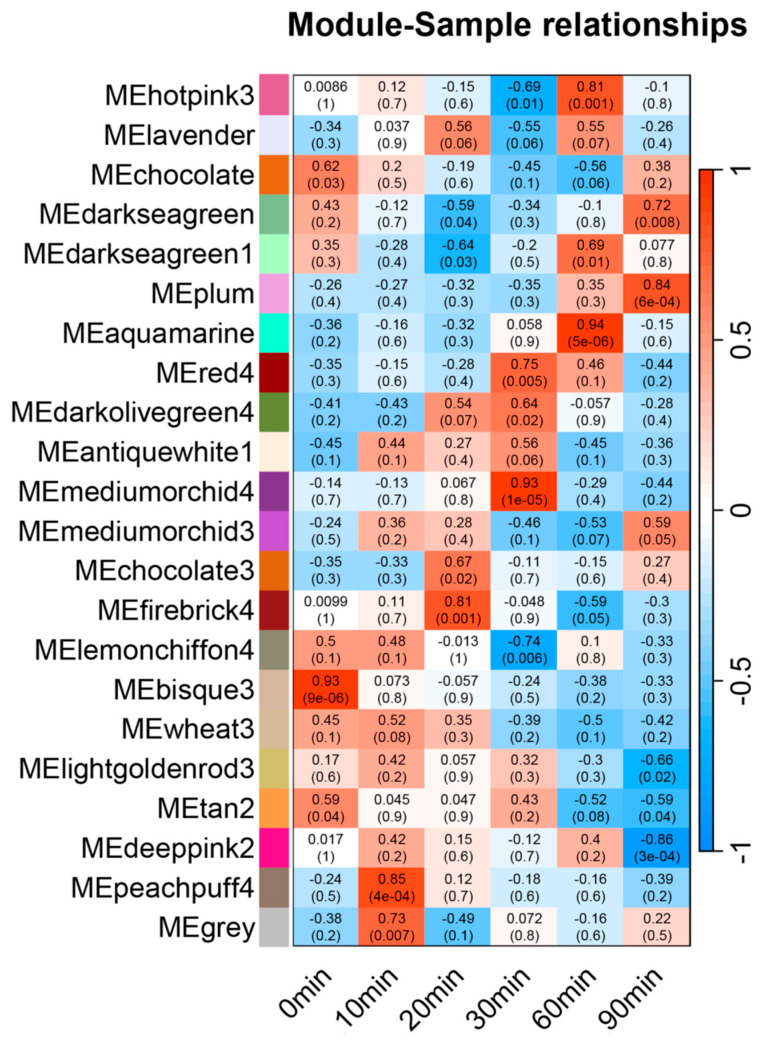
Nitrogen response module analysis of nitrate treatment at different times. Note: The expression patterns of the 22 co-expression modules obtained from the analysis over time are as follows: the horizontal axis represents different sampled time points, and the vertical axis represents gene modules. The grid color of each time point is red, representing positive correlation, and blue, representing negative correlation.

In order to explore the differences in expression patterns of various genes among differentially expressed genes and the co-expression relationship between transcription factors and functional genes, we constructed the WGCNA differential co-expression network. The genes with low expression were filtered according to TPM values, resulting in 22,768 high-expression genes. According to the feature vector of each module, the modules that are close to each other are combined to finally obtain 22 modules. Each module is represented by a different color (Figure 4). The results are shown in Figure 4. Among them, the plum module (*p*-value = 5 × 10^−4^, *r* = 0.84), aquamarine module (*p*-value = 5 × 10^−6^; *r* = 0.94), mediumorchid4 module (*p*-value = 1 × 10^−5^; *r* = 0.93), and peachpuff4 module (*p*-value = 4 × 10^−4^; *r* = 0.85) showed high positive correlation at 90 min, 60 min, 30 min, and 10 min, respectively. Meanwhile, the deeppink2 module (*p*-value = 3 × 10^−4^; *r* = −0.86) showed a strong negative correlation at 90 min. GO enrichment analysis was carried out on the module genes obtained above, revealing that three main modules are involved in nitrogen metabolism and regulation: aquamarine, mediumorchid4, and deeppink2 (Table 4).

### 3.4. Identification of Candidate Genes with Significant Nitrogen Uptake Traits

We analyzed the transcriptome data and found a total of 7744 differentially expressed genes at six time points. The most significantly differentially expressed 1685 genes were enriched into peachpuff4 and deeppink2 modules at the 90 min treatment. Through co-expression network analysis, it was found that among the 54 candidate genes closely linked to markers in GWAS and localization results, 17 genes (Table 5) were enriched in the aquamarine module. Four stable candidate genes that were repeatedly detected in single and multiple environments were *Zm00001d029012* (chr1: 54929627), *Zm00001d034035* (chr1: 281763909), *Zm00001d007890* (chr2: 241882616), and *Zm00001d045097* (chr9: 12398673). Among them, *Zm00001d045097* is a differentially expressed gene at the 60 min treatment and is up-regulated by nitrogen induction with a relative expression of 2.4. The gene annotation is multidrug resistance-associated protein 11. *Zm00001d029012*’s gene annotation is leucine-rich repeat protein kinase family protein. *Zm00001d034035*’s gene annotation is gsht1 and glutathione transporter1. *Zm00001d007890*’s gene annotation is YT521-B-like family protein. These four genes may have a strong correlation with the absorption and transport metabolism of nitrate in maize.

## 4. Discussion

### 4.1. GWAS Was Used to Screen the Candidate Genes Related to Nitrogen Uptake Traits

Using high heritability, scientific stability, abundant genetic variation, and a simple and economical index to measure crop nitrogen efficiency is the key to breeding nitrogen efficient germplasm. In this study, nitrate content was selected as the phenotypic index for GWAS analysis for the following reasons: First, maize is a dryland crop, and its primary form of nitrogen is nitrate. Second, nitrate is commonly used as an important index to reflect the differences in nitrogen accumulation in crops. The accumulation of nitrate in leaves can effectively indicate the plant’s nitrate absorption. Although other processes, such as metabolic assimilation, also affect the accumulation of nitrate forms in plants, the differences in nitrate uptake by different plants cannot be completely eliminated. The results of this study showed that nitrate content in leaves of 534 waxy maize germplasm resources showed good normal distribution characteristics, indicating that nitrate as a phenotypic index of GWAS analysis was scientific and representative. Eight concentration gradients of 0, 20, 40, 60, 80, 100, 140, and 200 (mg/L) were designed as references, and the nitrate content was calculated according to the trend line formula y = 0.0043x + 0.0051, and R^2^ = 0.9982 of the standard curve. There are two primary reasons why we chose Nanjing and Hainan to plant the 534 varieties of waxy corn. First, Nanjing is one of the main producing areas of waxy corn in the middle and lower reaches of the Yangtze River. Second, there is a significant difference in temperature between Nanjing and Hainan. Nanjing has a subtropical monsoon climate, with an average annual precipitation of about 1200 mm and an average annual temperature of 15.4 °C. In contrast, Hainan has a tropical monsoon climate, with an average annual precipitation of about 1640 mm and an average annual temperature of 22–26 °C. Even during the coldest months of January and February, the temperature in Hainan remains between 16 and 21 °C. Some studies have shown that high-temperature stress has become one of the main abiotic stresses affecting the yield and quality of waxy maize [40]. In this study, four candidate genes related to nitrogen absorption traits were co-located in both Nanjing and Hainan through GWAS. These genes were found to be little affected by temperature and exhibited stable genetic effects.

At present, candidate genes for nitrogen-use efficiency in barley have been identified through GWAS [41]. The whole genome was used to reveal the crucial role of the *BnaA8.ATG8F* gene in allotetraploid rapeseed adaptation to nitrogen restriction [42]. Multiple rice N-efficient gene loci, such as *OsTCP19*, *OsNAC42*, *OsNPF61*, *OsNLP4*, and *OsNiR*, have been identified based on GWAS [43]. The identification of these gene loci laid the foundation for construction of a molecular regulatory network for efficient nitrogen use. Many of these genes have been utilized in genetic improvement of efficient nitrogen use in modern cultivated rice. In this study, GWAS technology was used to discover 97 SNPs significantly associated with nitrate content in maize, involving 54 candidate genes, among which the SNP loci on chromosome 1 were the most widely distributed, with 5 candidate genes. *Zm00001d029012* and *Zm00001d034035* are two major genes on chromosome 1 that are closely related to nitrogen uptake traits at the seedling stage of maize. Previous studies reported that QTNs and candidate genes related to grain yield per plant (GYP), grain width (GW), kernel number per row (KNR), and tassel branch number (TBN) were located on chromosome 1 of maize using GWAS analysis [44]. On chromosome 2, we found a major locus, S2_241882616 (*Zm00001d007890*), that was significantly associated with the nitrogen uptake trait. In previous studies, 28 genes with 65 SNP loci were identified on chromosome 2 through GWAS analysis, many of which are related to the accumulation of trace elements in corn grains [45]. The gene we discovered is highly likely to promote the accumulation of nitrogen in corn grains and further affect the nutrition and quality of corn grains. The *Zm00001d045097* gene found at the main effect site of chromosome 9 (S9_12398673) is a multidrug resistance-associated protein 11. Previous studies have found QTL sites highly correlated with amylopectin content on maize’s ninth stain through GWAS localization [46]. The candidate gene *Zm00001d045097* that we discovered may regulate the nitrogen absorption of maize and thus affect the taste of waxy maize, which is worthy of further study. The candidate genes related to nitrogen absorption excavated in this study were derived from waxy maize materials. We took into account that waxy maize originated in China and has a long cultivation history, and there may be some ancient genes lost in common maize during the long domestication process.

### 4.2. Application of WGCNA Co-Expression Network in Candidate Gene Mining

At present, there are many reports on variety cultivation, yield improvement, industrial development, nutrient quality utilization, and taste improvement of waxy corn [2], but there are few studies on the molecular mechanism of how nitrogen affects the formation of yield and quality traits of waxy corn. In this study, candidate genes regulating nitrogen uptake in maize were studied by constructing a co-expression network. In recent years, gene function has been based on a large number of RNA sequencing data to construct co-expression gene networks of different plants, and the genes in the cluster module have the same expression pattern in different tissue development stages to predict [47]. It has been reported that conserved gene regulation patterns and related mechanisms have been explored in maize and rice, and in maize and sorghum through comparative analysis of gene networks [48]. However, the many, including disorderly, modules obtained by clustering in this way are usually not conducive to our screening of target traits. In this study, nitrate treatment was carried out at different time points at maize seedling stage according to maize nitrogen absorption traits, and finally three modules involved in nitrogen metabolism and regulation were obtained, so that we could screen candidate genes for maize nitrogen absorption more quickly.

In this study, it was found that among the 17 candidate genes identified for nitrogen uptake traits by combined GWAS and co-expression network analysis, 5 were located on chromosome 1. Among them, *Zm00001d029012* belonged to the class of leucine-rich repetitive receptor protein kinase family proteins. According to previous studies, *Zm00001d031678* is a gene that undergoes transcription subtype conversion specific to drought conditions in maize male ear, as enriched by GO [49]. *Zm00001d032578* is related to starch content in corn grains and is a candidate gene with transcription factor function [50]. *Zm00001d033159* is related to ear size, ear row number, and row number of grains in maize [51], indicating that these genes are related to maize yield traits. It is predicted that waxy corn may also indirectly impact nitrogen uptake by maize to improve maize yield. *Zm00001d034035* is a type of glutathione transporter that may be responsible for transporting nitrogen in maize. *Zm00001d007890*, located on chromosome 2, is a protein that interacts with corn-starch synthetase ZmSSIV, a class 521-B family protein [52]. There are two candidate genes on chromosome 3, one of which is the function of the *Zm00001d041638* gene, whose function is still unclear. *Zm00001d044300* has been analyzed in the protein interaction network, and this gene may play an important role in the cold-tolerance mechanism of maize [53,54]. The expression level of this gene is significantly high in transcriptome sequencing. As a candidate gene for cold-germination tolerance, it is possible that this gene’s role in the cold-tolerance mechanism of maize leads to strong plant metabolism and accelerated nitrogen uptake. The *Zm00001d014108* gene on chromosome 5 is a recombinant gene among 679 genes that have integrated into the population genome, influencing the expression variation in Teosinte [55]. This suggests that this gene may have been lost in common maize following the extended domestication process of Teosinte. Therefore, this gene is likely to be found only in waxy corn, where the most original genetic information is present. The function of *Zm00001d038109* in the two genes on chromosome 6 is still unclear. *Zm00001d038905* showed significantly down-regulated gene expression during the maize R1 period, indicating that it may have a negative regulatory function. In response to drought stress in maize, gene expression was significantly up-regulated in leaf tissues at 5 and 10 h after PEG8000 treatment, indicating a male-specific expression pattern [56]. The down-regulated expression of this gene in maize under drought stress may also promote nitrogen uptake. The functions of two out of the four candidate genes on chromosome 7, *Zm00001d021167* and *Zm00001d022414*, are still unclear. According to previous studies, *Zm00001d020501* is a nitric oxide reductase enzyme [57]. *Zm00001d021877* gene is a candidate gene significantly related to maize yield [58]. Based on the previous two studies, it is speculated that the *Zm00001d020501* gene plays a role in the process of reducing nitrate ions for nitrogen uptake in waxy corn. The *Zm00001d021877* gene promotes the accumulation of organic matter to increase the yield of waxy corn. The functions of *Zm00001d045097* on chromosome 9 and *Zm00001d025136* on chromosome 10 have not been mentioned in previous studies.

### 4.3. Analysis of Common Gene Function Discovered by GWAS and WGCNA

In this study, four candidate genes associated with nitrogen-uptake traits were identified through GWAS and nitrogen-response co-expression network analysis: *Zm00001d029012*, *Zm00001d034035*, *Zm00001d007890*, and *Zm00001d045097*. The first three showed a strong association with nitrogen metabolism. Previous studies have found that leucine-rich repeat receptor protein kinase (LRR-RLK) plays an important role in plant growth and development, pathogen defense, and environmental adaptation. A total of 467 LRR-RLK genes have been identified in legumes known for their strong nitrogen fixation ability [59]. *Zm00001d029012* belongs to the leucine-rich repetitive receptor protein kinase family, speculated to play a role in nitrogen metabolism and impact the growth and development of waxy corn. Previous studies have shown that glutathione and ATP-binding cassette (ABC) transporters of mitochondria (Atm) in *Arabidopsis* thaliana (AtAtm3) are associated with the maturation of cytoplasmic ferritin and heavy-metal detoxification [60]. It is speculated that the glutathione transporter *Zm00001d034035* is involved in glutamate cycling in maize, thereby promoting nitrogen accumulation in maize. *Zm00001d007890* is a 521-B family protein that interacts with corn-starch synthetase ZmSSIV. This interaction may play a role in the fixation of CO_2_ in the Calvin cycle, promote carbon and nitrogen balance, and ultimately impact the biological yield of waxy corn. These three candidate genes have great potential for nitrogen uptake and provide a new research direction for further studying the nitrogen-uptake traits of waxy corn.

## 5. Conclusions

In this study, a total of 534 waxy corn germplasm resources that had undergone deep sequencing were combined with 1,768,120 high-quality SNP markers to conduct a genome-wide association analysis on the nitrate content in aboveground parts of plants. We identified 97 SNPs significantly associated with nitrate-content traits and predicted 54 candidate genes. In total, 4 of the 17 candidate genes repeatedly located in multiple environments by GWAS were also successfully identified in co-expression network analysis. Four stable candidate genes, *Zm00001d029012* (chr1:54929627), *Zm00001d034035* (chr1:281763909), *Zm00001d007890* (chr2:241882616), and *Zm00001d045097* (chr9:12398673), were discovered as new genes related to nitrogen metabolism that have not been previously reported. The significant associated SNP sites and genes obtained in this study may play an important role in regulating nitrogen uptake and metabolism. However, the functional verification and breeding utilization of the identified genes need to be further explored, and the relevant in-depth research will be an important reference value for improving nitrogen-use efficiency of waxy corn through molecular-assisted breeding.

## Figures and Tables

**Table 1:** Data analysis of nitrate content (mg/kg) in local waxy corn in a single environment or multiple environments.

Year	Sample Size	Minimum	Maximum	Mean	Median	Standard Deviation	Variance	Skewness	Kurtosis
2021	534	437.86	3741.43	2025.22	2130.71	785.67	617,280.64	−0.400	−0.603
2022	534	457.46	3781	2030.19	2120.21	782.42	612,179.10	−0.354	−0.572
Blup	534	−1402.12	1538.34	0.0000	86.8067	695.78	484,109.79	−0.378	−0.588

**Table 2:** Whole-genome variation information of waxy corn.

Chromosome	Length (bp)	Variance
chr1	301,354,135	16,408,388
chr2	237,068,873	7,902,973
chr3	232,140,174	12,809,543
chr4	241,473,504	12,354,858
chr5	217,872,852	12,993,741
chr6	169,174,353	11,076,019
chr7	176,764,762	8,638,318
chr8	175,793,759	9,368,327
chr9	156,750,706	9,214,786
chr10	150,189,435	8,411,799
**Total**	**2,058,582,553**	**109,178,752**

**Table 3:** SNP information associated with trait stability in single or multiple environments.

Chromosome	Position (bp)	Trait Name	LOD	r2 (%)	*p*-Value	GeneID
1	54,929,627	E1	8.13	1.94	9.46 × 10^−10^	***Zm00001d029012***
		multi_env	8.42	0.16	4.81 × 10^−10^	
	92,818,447	E1	6.40	2.62	5.64 × 10^−8^	
		E2	8.46	3.31	4.30 × 10^−10^	
		multi_env_BLUP	9.38	4.02	5.00 × 10^−11^	
	198,532,332	E2	8.11	2.29	9.95 × 10^−10^	*Zm00001d031678*
		multi_env_BLUP	4.47	1.34	5.67 × 10^−6^	
		multi_env	9.25	0.22	6.65 × 10^−11^	
	231,151,343	E1	5.97	1.91	1.57 × 10^−7^	*Zm00001d032578*
		E2	10.51	3.27	3.46 × 10^−12^	
		multi_env_BLUP	8.46	2.83	4.36 × 10^−10^	
		multi_env	7.79	0.20	2.09 × 10^−9^	
	252,525,197	E1	7.89	4.31	1.66 × 10^−9^	*Zm00001d033159*
		E2	5.75	2.92	2.65 × 10^−7^	
		multi_env_BLUP	10.11	5.77	8.90 × 10^−12^	
	281,763,909	E1	14.25	3.60	5.42 × 10^−16^	***Zm00001d034035***
		E2	13.56	3.21	2.71 × 10^−15^	
		multi_env_BLUP	19.26	5.17	4.62 × 10^−21^	
		multi_env	29.41	0.60	2.67 × 10^−31^	
	286,796,425	E1	7.47	3.56	4.54 × 10^−9^	
		E2	7.85	3.55	1.81 × 10^−9^	
		multi_env_BLUP	6.08	2.96	1.22 × 10^−7^	
2	241,882,616	E1	11.91	1.88	1.32 × 10^−13^	***Zm00001d007890***
		multi_env_BLUP	9.80	1.57	1.85 × 10^−11^	
		multi_env	34.07	0.45	5.40 × 10^−36^	
3	105,062,481	E1	9.28	1.22	6.30 × 10^−11^	
		E2	10.29	1.29	5.84 × 10^−12^	
		multi_env	64.55	0.81	1.31 × 10^−66^	
	130,776,903	E1	16.58	3.13	2.38 × 10^−18^	*Zm00001d041638*
		E2	16.10	2.86	7.32 × 10^−18^	
		multi_env_BLUP	18.01	3.53	8.53 × 10^−20^	
		multi_env	97.67	1.88	8.16 × 10^−100^	
	224,502,110	E1	10.01	2.28	1.12 × 10^−11^	*Zm00001d044300*
		E2	13.14	2.88	7.32 × 10^−15^	
		multi_env_BLUP	14.21	3.42	6.05 × 10^−16^	
5	32,772,711	E1	6.04	2.01	1.33 × 10^−7^	*Zm00001d014108*
		multi_env_BLUP	9.06	3.16	1.04 × 10^−10^	
	75,859,584	E2	5.43	1.35	5.76 × 10^−7^	
		multi_env	89.83	2.52	5.88 × 10^−92^	
	111,565,300	E1	13.54	2.76	2.86 × 10^−15^	
		E2	11.65	2.21	2.38 × 10^−13^	
		multi_env_BLUP	11.50	2.38	3.45 × 10^−13^	
6	148,266,713	E1	7.48	1.92	4.33 × 10^−9^	*Zm00001d038109*
		E2	10.14	2.50	8.39 × 10^−12^	
		multi_env	22.24	0.47	4.54 × 10^−24^	
	166,762,568	E1	12.17	3.46	7.10 × 10^−14^	*Zm00001d038905*
		E2	12.34	3.32	4.79 × 10^−14^	
		multi_env_BLUP	11.90	3.48	1.33 × 10^−13^	
		multi_env	14.49	0.32	3.14 × 10^−16^	
7	3,775,500	E1	7.72	3.31	2.47 × 10^−9^	
		E2	7.95	3.23	1.43 × 10^−9^	
		multi_env_BLUP	7.17	3.16	9.12 × 10^−9^	
	119,463,838	E1	6.09	3.33	1.20 × 10^−7^	*Zm00001d020501*
		E2	8.98	4.72	1.26 × 10^−10^	
		multi_env_BLUP	7.50	4.25	4.21 × 10^−9^	
		multi_env	13.16	0.59	7.02 × 10^−15^	
	144,661,743	E1	6.99	2.08	1.40 × 10^−8^	*Zm00001d021167*
		E2	5.68	1.58	3.14 × 10^−7^	
		multi_env	57.17	1.60	3.32 × 10^−59^	
	165,275,579	E2	9.90	1.58	1.46 × 10^−11^	*Zm00001d021877*
		multi_env_BLUP	7.83	1.34	1.91 × 10^−9^	
		multi_env	19.23	0.26	4.94 × 10^−21^	
	177,122,793	E1	9.29	5.23	6.08 × 10^−11^	*Zm00001d022414*
		E2	7.44	3.90	4.83 × 10^−9^	
		multi_env_BLUP	5.84	3.30	2.17 × 10^−7^	
8	70,304,376	E1	26.49	5.15	2.34 × 10^−28^	
		E2	23.36	4.19	3.32 × 10^−25^	
		multi_env_BLUP	22.44	4.36	2.85 × 10^−24^	
		multi_env	15.29	0.21	4.83 × 10^−17^	
9	12,398,673	E1	6.55	2.47	3.99 × 10^−8^	***Zm00001d045097***
		E2	6.21	2.20	8.99 × 10^−8^	
		multi_env_BLUP	8.89	3.50	1.59 × 10^−10^	
		multi_env	14.56	0.45	2.63 × 10^−16^	
10	106,059,138	E1	5.58	2.98	4.02 × 10^−7^	*Zm00001d025136*
		multi_env_BLUP	4.73	2.58	3.08 × 10^−6^	

**Table 4:** GO enrichment items of nitrogen response module genes.

ME Names	Main BP	Gene No.	FDR
Aquamarine	Nitrogen compound metabolic process	1789/5780	3.1 × 10^−17^
Mediumorchild4	Regulation of nitrogen compound metabolic process	66/388	0.0014
Deeppink2	Nitrogen compound metabolic process	764/2556	5.6 × 10^−5^
Peachpuff4	Cellular macromolecule metabolic process	13/37	0.04
Plum	Single-organism process	1512/1881	2.2 × 10^−9^

**Table 5:** Candidate genes and functional annotation.

Markers	Gene Name	Chromosome	*p*-Value	Gene Annotation
S1_54929627	***Zm00001d029012***	1	4.81 × 10^−10^	Leucine-rich repeat protein kinase family protein
S1_198532332	*Zm00001d031678*	1	6.65 × 10^−11^	rrb3; retinoblastoma family3
S1_231151343	*Zm00001d032578*	1	2.09 × 10^−9^	Dof zinc finger protein DOF1.6
S1_252525197	*Zm00001d033159*	1	8.90 × 10^−12^	DEK domain-containing chromatin associated protein
S1_281763909	***Zm00001d034035***	1	2.67 × 10^−31^	gsht1; glutathione transporter1
S2_241882616	***Zm00001d007890***	2	5.40 × 10^−36^	YT521-B-like family protein
S3_130776903	*Zm00001d041638*	3	8.16 × 10^−100^	
S3_224502110	*Zm00001d044300*	3	6.05 × 10^−16^	
S5_32772711	*Zm00001d014108*	5	5.88 × 10^−92^	uce8; ubiquitin conjugating enzyme 8
S6_148266713	*Zm00001d038109*	6	4.54 × 10^−24^	
S6_166762568	*Zm00001d038905*	6	3.14 × 10^−16^	Probable β-14-xylosyltransferase IRX10L
S7_119463838	*Zm00001d020501*	7	7.02 × 10^−15^	RING/U-box superfamily protein
S7_144661743	*Zm00001d021167*	7	3.32 × 10^−59^	UDP-glycosyltransferase 74B1
S7_165275579	*Zm00001d021877*	7	4.94 × 10^−21^	ak1; adenylyl-sulfate kinase 1
S7_177122793	*Zm00001d022414*	7	2.17 × 10^−7^	Ubiquitin carboxyl-terminal hydrolase 24
S9_12398673	***Zm00001d045097***	9	2.63 × 10^−16^	Multidrug resistance-associated protein 11
S10_106059138	*Zm00001d025136*	10	3.08 × 10^−6^	

## Data Availability

The original contributions presented in this study are included in the article/Supplementary Materials. Further inquiries can be directed to the corresponding author.

## Supplementary Materials

The following supporting information can be downloaded at https://www.mdpi.com/article/10.3390/genes16020126/s1, Supplementary Table S1: A list of 534 local waxy corn germplasm resources.
