# Supplementary material for: Integration of GWAS and Co-Expression Network Analysis Identified Main Genes Responsible for Nitrogen Uptake Traits in Seedling Waxy Corn
Source: Genes (Basel). 2025 Jan 23;16(2):126. doi: 10.3390/genes16020126 (PMC11854815; doi:10.3390/genes16020126)
Supplement: Supplementary file 1 [file genes-16-00126-s001.zip › genes-3378053-supplementary.pdf]

Sequencing number	Material name	Sequencing number	Material name
JAAS001	local waxy corn	JAAS043	DN14
JAAS002	19-WS740	JAAS044	DN17
JAAS003	S347	JAAS045	DN24
JAAS004	S21-ZW004	JAAS046	DN33
JAAS005	S21-ZW005	JAAS047	N269
JAAS006	S21-ZW006	JAAS048	N271
JAAS007	Huaiyin purple waxy corn	JAAS049	N320
JAAS008	Heng bai522	JAAS050	N388
JAAS009	Haimen purple waxy corn	JAAS051	ZW4001
JAAS010	local waxy corn	JAAS052	Guanglingxiang waxy corn
JAAS011	S21-ZW011	JAAS053	1-101
JAAS012	CN14791	JAAS054	HW315
JAAS013	DN1012	JAAS055	1-124
JAAS014	DN830	JAAS056	Caihua waxy corn
JAAS015	JDN003	JAAS057	AF08A-0903-79
JAAS016	N27	JAAS058	Qx0323P
JAAS017	CN14791	JAAS059	M3A00804897
JAAS018	S181	JAAS060	SHWL02
JAAS019	W41	JAAS061	SHWL03
JAAS020	BN08	JAAS062	SHWL04
JAAS021	M3A00804895	JAAS063	SHWL06
JAAS022	local waxy corn	JAAS064	SHWL07
JAAS023	WX004	JAAS065	SHWL08
JAAS024	Guangxi N4	JAAS066	SHWL09
JAAS025	Haimen purple corn	JAAS067	SHWL11
JAAS026	Jingnuo 210	JAAS068	M3A00804898
JAAS027	local waxy corn	JAAS069	M3A00804899
JAAS028	2016N9024	JAAS070	WN25
JAAS029	N42-17	JAAS071	QW04
JAAS030	local waxy corn	JAAS072	Dan79
JAAS031	N9	JAAS073	local waxy corn
JAAS032	Sichuan purple waxy corn	JAAS074	local waxy corn
JAAS033	N08	JAAS075	local waxy corn
JAAS034	local waxy corn	JAAS076	local waxy corn
JAAS035	S21-ZW035	JAAS077	local waxy corn
JAAS036	local waxy corn	JAAS078	local waxy corn
JAAS037	Luo5	JAAS079	19-Zw142
JAAS038	M3A00804896	JAAS080	S388
JAAS039	T361	JAAS081	CN13-1301
JAAS040	local waxy corn	JAAS082	CN13-1302
JAAS041	local waxy corn	JAAS083	CN13-1304
JAAS042	local waxy corn	JAAS084	CN13-1305

Sequencing number	Material name	Sequencing number	Material name
JAAS085	CN13-1307	JAAS127	SHWL12
JAAS086	CN13-1308	JAAS128	SHWL16
JAAS087	M3A00804901	JAAS129	M3A00804633
JAAS088	M3A00804902	JAAS130	local waxy corn
JAAS089	CN13-1332	JAAS131	WN92
JAAS090	CN13-1334	JAAS132	local waxy corn
JAAS091	CN13-1339	JAAS133	local waxy corn
JAAS092	CN13-1342	JAAS134	BN08
JAAS093	CN13-1343	JAAS135	CHN-12-44
JAAS094	DN75668-1	JAAS136	HN3
JAAS095	DN75668-2	JAAS137	M3A00804905
JAAS096	W18	JAAS138	2017N9016
JAAS097	H366	JAAS139	local waxy corn
JAAS098	19-Zw304	JAAS140	M3A00804906
JAAS099	H-146	JAAS141	local waxy corn
JAAS100	H09097	JAAS142	local waxy corn
JAAS101	8P091	JAAS143	local waxy corn
JAAS102	BW1306	JAAS144	local waxy corn
JAAS103	hw1401	JAAS145	M3A00804907
JAAS104	NFRF	JAAS146	M3A00804908
JAAS105	361	JAAS147	W8N008
JAAS106	JDWM	JAAS148	19-Zw291
JAAS107	ke1-8	JAAS149	local waxy corn
JAAS108	HDWM	JAAS150	N15
JAAS109	local waxy corn	JAAS151	WN27
JAAS110	local waxy corn	JAAS152	CN13-1300
JAAS111	Changzhou small yellow corn	JAAS153	CN13-1303
JAAS112	Golden waxy corn	JAAS154	CN13-1306
JAAS113	local waxy corn	JAAS155	S19-WS631
JAAS114	local waxy corn	JAAS156	CN13-1310
JAAS115	East purple waxy corn	JAAS157	CN13-1311
JAAS116	local waxy corn	JAAS158	M3A00804911
JAAS117	local waxy corn	JAAS159	CN13-1314
JAAS118	local waxy corn	JAAS160	M3A00804912
JAAS119	local waxy corn	JAAS161	CN13-1318
JAAS120	NM	JAAS162	CN13-1319
JAAS121	w6	JAAS163	CN13-1322
JAAS122	M3A00804904	JAAS164	CN13-1329
JAAS123	N071	JAAS165	CN13-1330
JAAS124	1-097	JAAS166	S19-WS647
JAAS125	SHWL05	JAAS167	CN13-1333
JAAS126	SHWL10	JAAS168	CN13-1335

Sequencing number	Material name	Sequencing number	Material name
JAAS169	CN13-1336	JAAS211	MVTC200
JAAS170	CN13-1337	JAAS212	IRR15
JAAS171	CN13-1338	JAAS213	Wuming 213
JAAS172	CN13-1340	JAAS214	Huadianbaibaomi
JAAS173	N44	JAAS215	local waxy corn
JAAS174	19-Zw275	JAAS216	local waxy corn
JAAS175	local waxy corn	JAAS217	local waxy corn
JAAS176	local waxy corn	JAAS218	local waxy corn
JAAS177	local waxy corn	JAAS219	local waxy corn
JAAS178	local waxy corn	JAAS220	local waxy corn
JAAS179	M3A00802346	JAAS221	local waxy corn
JAAS180	M3A00802353	JAAS222	Multi-ear corn
JAAS181	M3A00802362	JAAS223	S21-ZW229
JAAS182	M3A00802365	JAAS224	Erhulutou
JAAS183	M3A00802367	JAAS225	S21-ZW231
JAAS184	M3A00802380	JAAS226	Xiaoqingke
JAAS185	M3A00802385	JAAS227	Gongbai 101
JAAS186	M3A00802392	JAAS228	Tielingbaimaya
JAAS187	M3A00802393	JAAS229	Huabatang
JAAS188	M3A00802395	JAAS230	Bolibaomi
JAAS189	M3A00802401	JAAS231	Huaidemianbaomi
JAAS190	M3A00802406	JAAS232	PR37
JAAS191	M3A00802412	JAAS233	S21-ZW239
JAAS192	M3A00802415	JAAS234	Shuangyanghongbaomi
JAAS193	M3A00802417	JAAS235	Huangtangbaomi
JAAS194	M3A00802418	JAAS236	TielinghuangmayaY
JAAS195	M3A00802423	JAAS237	S21-ZW243
JAAS196	M3A00802429	JAAS238	S21-ZW244
JAAS197	M3A00802431	JAAS239	S21-ZW245
JAAS198	M3A00802432	JAAS240	Fengchengbaitoushuang
JAAS199	M3A00802448	JAAS241	Synthetic Early dent
JAAS200	M3A00802454	JAAS242	HOH792
JAAS201	M3A00802455	JAAS243	Cang
JAAS202	M3A00802456	JAAS244	Cangiceneal
JAAS203	M3A00802478	JAAS245	Hp21whitefintbybnid
JAAS204	M3A00802479	JAAS246	DF28Flintdenthybrid
JAAS205	M3A00802481	JAAS247	″oh45Model″syn
JAAS206	Spikelet yellow	JAAS248	IongFellow
JAAS207	Nonganbaibatang	JAAS249	Jia 240
JAAS208	Golden summit	JAAS250	OVari3sz
JAAS209	S21-ZW215	JAAS251	S21-ZW257
JAAS210	Changpin 687	JAAS252	local waxy corn

Sequencing number	Material name	Sequencing number	Material name
JAAS253	S21-ZW259	JAAS295	Core corn
JAAS254	local waxy corn	JAAS296	local waxy corn
JAAS255	local waxy corn	JAAS297	local waxy corn
JAAS256	local waxy corn	JAAS298	Yellow glutinous rice
JAAS257	S21-ZW263	JAAS299	Yellow sticky corn
JAAS258	Xiaolihong-4	JAAS300	Yuer corn
JAAS259	Huaidejinding purple waxy corn	JAAS301	Sticky stick
JAAS260	Dunhuabaitoushuang	JAAS302	NF
JAAS261	Tonghuabaitoushuang	JAAS303	Szw
JAAS262	Tonghuadaqingke	JAAS304	local waxy corn
JAAS263	S21-ZW269	JAAS305	local waxy corn
JAAS264	Lishuhuobaomi	JAAS306	Wubaogu
JAAS265	Xiaobaici	JAAS307	local waxy corn
JAAS266	Nonganbaimaya	JAAS308	DN15
JAAS267	Baimaya	JAAS309	N327
JAAS268	Fangzibaimaya	JAAS310	M3A00804923
JAAS269	Sanchahebaimaya	JAAS311	M3A00804924
JAAS270	Jidongbaimaya	JAAS312	M3A00804925
JAAS271	Baihenian	JAAS313	M3A00804926
JAAS272	S21-ZW278	JAAS314	M3A00804927
JAAS273	Weihulingbaibaomi	JAAS315	Jingnuo 6
JAAS274	19-Zw087	JAAS316	RK1493
JAAS275	19-Zw185	JAAS317	Old white waxy corn
JAAS276	19-Zw230	JAAS318	RK1765
JAAS277	19-Zw243	JAAS319	RK1766
JAAS278	19-Zw416	JAAS320	RK1793
JAAS279	S19-WS634	JAAS321	RK1797
JAAS280	S19-WS635	JAAS322	RK1801
JAAS281	S19-WS637	JAAS323	RK1802
JAAS282	S19-WS642	JAAS324	RK1803
JAAS283	S19-WS643	JAAS325	RK1804
JAAS284	Jiaxin waxy corn	JAAS326	RK1808
JAAS285	S21-ZW291	JAAS327	RK1812
JAAS286	S21-ZW292	JAAS328	RK1813
JAAS287	S21-ZW293	JAAS329	Small corn
JAAS288	Yangbangtou	JAAS330	RK1864
JAAS289	Small sticky stick head	JAAS331	RK1865
JAAS290	Yuer corn	JAAS332	Waxy Jade's strength
JAAS291	Twodaysofrough waxy corn	JAAS333	Nubaogu
JAAS292	Small white waxy corn	JAAS334	RK1921
JAAS293	White waxy corn	JAAS335	RK1925
JAAS294	Waxy corn in Deer Garden	JAAS336	RK1974

Sequencing number	Material name	Sequencing number	Material name
JAAS337	RK1999	JAAS379	RK1874
JAAS338	M3A00802462	JAAS380	local waxy corn
JAAS339	M3A00802477	JAAS381	local waxy corn
JAAS340	local waxy corn	JAAS382	RK1894
JAAS341	Huayu	JAAS383	RK1896
JAAS342	Hai sheng	JAAS384	RK1899
JAAS343	S21-ZW352	JAAS385	RK1900
JAAS344	S21-ZW353	JAAS386	RK1933
JAAS345	S21-ZW354	JAAS387	M3A00802382
JAAS346	S21-ZW355	JAAS388	RK1953
JAAS347	Shi luo	JAAS389	M3A00802403
JAAS348	S21-ZW357	JAAS390	RK1976
JAAS349	S21-ZW358	JAAS391	RK1990
JAAS350	S21-ZW359	JAAS392	M3A00802438
JAAS351	Luyuan Glutinous Corn	JAAS393	M3A00802457
JAAS352	local red waxy corn	JAAS394	M3A00802458
JAAS353	Little Yankee head	JAAS395	M3A00802484
JAAS354	Black glutinous corn	JAAS396	M3A00802487
JAAS355	Small yellow corn	JAAS397	RK2052
JAAS356	Small purple corn	JAAS398	RK2053
JAAS357	M3A00804946	JAAS399	RK2054
JAAS358	Luyuan Glutinous Corn	JAAS400	RK2055
JAAS359	local waxy corn	JAAS401	RK2057
JAAS360	local waxy corn	JAAS402	RK2058
JAAS361	local waxy corn	JAAS403	RK2059
JAAS362	RK2148	JAAS404	RK2060
JAAS363	local waxy corn	JAAS405	local waxy corn
JAAS364	local waxy corn	JAAS406	local waxy corn
JAAS365	RK2158	JAAS407	RK2138
JAAS366	RK1488	JAAS408	RK2155
JAAS367	RK1491	JAAS409	RK1945
JAAS368	RK1768	JAAS410	local waxy corn
JAAS369	RK1769	JAAS411	local waxy corn
JAAS370	Shennongnuo	JAAS412	local waxy corn
JAAS371	RK1790	JAAS413	local waxy corn
JAAS372	Guangxiliu	JAAS414	local waxy corn
JAAS373	RK1809	JAAS415	local waxy corn
JAAS374	Yunnanhong	JAAS416	M3A00804841
JAAS375	RK1867	JAAS417	M3A00804842
JAAS376	Hongliangzhan	JAAS418	M3A00804843
JAAS377	local waxy corn	JAAS419	local waxy corn
JAAS378	Tongzhan	JAAS420	local waxy corn

Sequencing number	Material name	Sequencing number	Material name
JAAS421	M3A00804975	JAAS463	J82 yellow N-3N-1
JAAS422	local waxy corn	JAAS464	J84-4-8
JAAS423	local waxy corn	JAAS465	19-Zw196
JAAS424	local waxy corn	JAAS466	M3A00804980
JAAS425	local waxy corn	JAAS467	19-Zw278
JAAS426	local waxy corn	JAAS468	19-Zw286
JAAS427	local waxy corn	JAAS469	M3A00804983
JAAS428	local waxy corn	JAAS470	local waxy corn
JAAS429	local waxy corn	JAAS471	local waxy corn
JAAS430	local waxy corn	JAAS472	RK1807
JAAS431	N4	JAAS473	RK1798
JAAS432	local waxy corn	JAAS474	Little yellow corn
JAAS433	local waxy corn	JAAS475	M3A00804986
JAAS434	local waxy corn	JAAS476	M3A00804987
JAAS435	Yellow sweet su	JAAS477	19-Zw092
JAAS436	local waxy corn	JAAS478	19-Zw093
JAAS437	N14	JAAS479	19-Zw113
JAAS438	waxy10	JAAS480	19-Zw117
JAAS439	M3A00804976	JAAS481	M3A00804992
JAAS440	Black315	JAAS482	19-Zw148
JAAS441	local waxy corn	JAAS483	19-Zw289
JAAS442	local waxy corn	JAAS484	19-Zw294
JAAS443	local waxy corn	JAAS485	19-Zw296
JAAS444	Jingtiannuo 2	JAAS486	M3A00804997
JAAS445	T1416	JAAS487	19-Zw338
JAAS446	local waxy corn	JAAS488	local waxy corn
JAAS447	local waxy corn	JAAS489	SHWL13
JAAS448	local waxy corn	JAAS490	M3A00804999
JAAS449	local waxy corn	JAAS491	S21-ZW517
JAAS450	JSC7002	JAAS492	Nantong purple waxy corn
JAAS451	M3A00804977	JAAS493	M3A00805000
JAAS452	local waxy corn	JAAS494	M3A00805001
JAAS453	M3A00804978	JAAS495	S21-ZW524
JAAS454	local waxy corn	JAAS496	M3A00805002
JAAS455	local waxy corn	JAAS497	M3A00805003
JAAS456	N310	JAAS498	M3A00805004
JAAS457	local waxy corn	JAAS499	M3A00805005
JAAS458	WT104	JAAS500	M3A00805006
JAAS459	local waxy corn	JAAS501	Accokp
JAAS460	J75	JAAS502	LG11
JAAS461	QN51	JAAS503	M3A00802441
JAAS462	J27-6N	JAAS504	M3A00805007

Sequencing number	Material name
JAAS505	prmaINRA186
JAAS506	RK1962
JAAS507	local waxy corn
JAAS508	M3A00804888
JAAS509	RK2144
JAAS510	S21-ZW540
JAAS511	S21-ZW541
JAAS512	M3A00805009
JAAS513	M3A00805010
JAAS514	M3A00805011
JAAS515	M3A00805012
JAAS516	M3A00805013
JAAS517	M3A00805014
JAAS518	M3A00805015
JAAS519	M3A00805016
JAAS520	M3A00805017
JAAS521	M3A00805018
JAAS522	M3A00805019
JAAS523	M3A00805020
JAAS524	M3A00805022
JAAS525	M3A00805023
JAAS526	M3A00805024
JAAS527	M3A00805025
JAAS528	M3A00805026
JAAS529	M3A00805027
JAAS530	M3A00805028
JAAS531	M3A00805029
JAAS532	M3A00805030
JAAS533	M3A00805031
JAAS534	M3A00805032
